# An Integrative Genomic and Transcriptomic Analysis Reveals Potential Targets Associated with Cell Proliferation in Uterine Leiomyomas

**DOI:** 10.1371/journal.pone.0057901

**Published:** 2013-03-04

**Authors:** Priscila Daniele Ramos Cirilo, Fábio Albuquerque Marchi, Mateus de Camargo Barros Filho, Rafael Malagoli Rocha, Maria Aparecida Custódio Domingues, Igor Jurisica, Anagloria Pontes, Silvia Regina Rogatto

**Affiliations:** 1 Department of Genetics, Institute of Biosciences, UNESP - São Paulo State University, Botucatu, São Paulo, Brazil; 2 CIPE - NeoGene Laboratory, AC Camargo Hospital, Fundação Antonio Prudente, São Paulo, São Paulo, Brazil; 3 Inter-institutional Grad Program on Bioinformatics, Institute of Mathematics and Statistics, USP – São Paulo University, São Paulo, São Paulo, Brazil; 4 Department of Anatomic Pathology, AC Camargo Hospital, Fundação Antonio Prudente, São Paulo, São Paulo, Brazil; 5 Department of Pathology, School of Medicine, UNESP - São Paulo State University, Botucatu, São Paulo, Brazil; 6 Ontario Cancer Institute, the Campbell Family Institute for Cancer Research, and Techna Institute, University Health Network, Toronto, Ontario, Canada; 7 Department of Gynaecology and Obstetrics, School of Medicine, São Paulo State University, Botucatu, São Paulo, Brazil; 8 Department of Urology, School of Medicine, UNESP - São Paulo State University, Botucatu, São Paulo, Brazil; Deutsches Krebsforschungszentrum, Germany

## Abstract

**Background:**

Uterine Leiomyomas (ULs) are the most common benign tumours affecting women of reproductive age. ULs represent a major problem in public health, as they are the main indication for hysterectomy. Approximately 40–50% of ULs have non-random cytogenetic abnormalities, and half of ULs may have copy number alterations (CNAs). Gene expression microarrays studies have demonstrated that cell proliferation genes act in response to growth factors and steroids. However, only a few genes mapping to CNAs regions were found to be associated with ULs.

**Methodology:**

We applied an integrative analysis using genomic and transcriptomic data to identify the pathways and molecular markers associated with ULs. Fifty-one fresh frozen specimens were evaluated by array CGH (JISTIC) and gene expression microarrays (SAM). The CONEXIC algorithm was applied to integrate the data.

**Principal Findings:**

The integrated analysis identified the top 30 significant genes (*P*<0.01), which comprised genes associated with cancer, whereas the protein-protein interaction analysis indicated a strong association between FANCA and BRCA1. Functional *in silico* analysis revealed target molecules for drugs involved in cell proliferation, including FGFR1 and IGFBP5. Transcriptional and protein analyses showed that FGFR1 (*P* = 0.006 and *P*<0.01, respectively) and IGFBP5 (*P* = 0.0002 and *P* = 0.006, respectively) were up-regulated in the tumours when compared with the adjacent normal myometrium.

**Conclusions:**

The integrative genomic and transcriptomic approach indicated that *FGFR1* and *IGFBP5* amplification, as well as the consequent up-regulation of the protein products, plays an important role in the aetiology of ULs and thus provides data for potential drug therapies development to target genes associated with cellular proliferation in ULs.

## Introduction

Uterine Leiomyomas (ULs) are the most common benign tumours in women of reproductive age and affect 25–30% of women [1]. ULs are smooth muscle tumours, and multiple tumours are often found in the same uterus [2]. Although they are extremely common and present an important public health problem, the biology of these tumours remains unexplained. Most of these tumours are asymptomatic, and only 25% of individuals have clinical symptoms, such as pelvic pain, abnormal bleeding, infertility and pregnancy complications [3]. Oestrogen and progesterone are the most critical regulators of fibroid growth [4], [5].

The deregulation of growth factors [6] and microRNAs (miRNAs) [7], shortening of telomeres [8], excessive production of disorganised extracellular matrix [6], [9], loss of heterozygosity [10], and recurrent chromosomal aberrations (for review, see [11]) have been suggested to contribute to the growth of fibroids. Cytogenetic studies (for review, see [12]) in ULs have demonstrated several recurrent chromosomal alterations, including deletions in 7q, trisomy of chromosome 12 and chromosomal rearrangements such as translocations involving the *HMGA2* gene [13]. Recently, Mäkinen et al. [14], using exome-sequencing, reported a tumour-specific mutation (exon 12) in the *MED12* gene in a large subgroup of ULs. Gene expression microarray studies revealed alterations involving mainly genes associated with cell proliferation, the cell cycle, differentiation and extracellular matrix production (for review, see [15]). Furthermore, using indirect correlation analysis, three studies have associated copy number alterations (CNAs) with gene expression deregulation in ULs [16], [17], [18].

There are currently no effective therapies available for ULs that are directed at molecular targets. The identification of driver genes (encoding modulator molecules) for tumorigenesis is a crucial challenge to identify new molecules for therapy. DNA copy number alteration is one of several events that can regulate gene expression [19] and consequently the protein products. Recently, studies using genomic and transcriptomic integrative analysis in cancer have identified driver genes [20], [21] that could be involved in the mechanisms of disease evolution and provided new potential candidates for therapeutic intervention [22].

In this study, we performed array CGH and large-scale expression analysis in 51 ULs from 34 patients. The data were integrated using the algorithm COpy Number and EXpression In Cancer CONEXIC [19]. In addition, the functional analysis of networks and canonical pathways of modulator molecules was applied to evaluate molecular pathways involved in ULs pathogenesis that could be useful for the selection of putative markers and for defining target therapies. Based on the results, we confirmed the involvement of the *FGFR1* and *IGFBP5* genes by real-time reverse transcription polymerase chain reaction (RT-qPCR) and their protein expression by immunohistochemistry (IHC) on a tissue microarray. In this integrative analysis, we provide new insights about the pathogenesis of ULs and identified candidate biomarkers for therapy in ULs.

## Results

### DNA Copy Number Alteration Analysis

Fifty-one ULs were hybridised to 44K Agilent arrays to determine copy number alterations. In total, 170 recurrent CNAs were detected, of which 142 regions had gains (1,192 genes) and 18 had losses (160 genes). The most frequent genomic imbalances were gains on chromosomes 16 (16p11.2, 16q22.1, 16q24.3) and 19 (19p13.3, 19q13.32, 19q13.32-q13.33) and losses on chromosomes 4 (4p14, 4q13.1, 4q28.3) and 16 (16p13.12-p13.11, 16p11.2-p11.1, 16q23.1) (Table S1). No significant correlation was found between specific regions with a CNA and the clinical data (data not shown).

### Gene Expression Analysis

Based on the unsupervised hierarchical clustering analysis of the gene expression data, the identification of subgroups of tumours according to the clinical features was not possible. The gene expression analysis identified 3,325 significant genes: 1,138 were up-regulated, and 2,187 were down-regulated.

### Integrative Analysis

The integrative analysis using CONEXIC revealed 1,192 up-regulated genes and 3,325 down-regulated genes with significant G-scores and ranked 75 modulators, which mapped to 1p36.13, 1q41, 2q32.1, 2q32.2, 2q35, 4p14, 5q31.2, 5q35.3, 7q22.1, 8p12, 8q24.3, 10p15.3, 10q21.3, 11p15.5, 11q13.2, 12p11.21, 12p13.31, 14q13.2, 16p11.2, 16q24.3, 17q21.31, 19q13.32, 19q13.33, 20p11.2 and 20p13 (Table 1).

**Table 1:** Seventy-five modulators obtained from integrative analysis.

CONEXIC Score	Array CGH	Expression Array	Region	Position Hg18 (Start-End)	Gene symbol
1	*+	−	5q31.2^£^	137,543,248–137,551,261	***KIF20A***
2	+	+		218,372,757–218,517,041	***TNS1***
3	+	+	1p36.13^£^	16,213,110–16,217,872	***HSPB7***
4	+	−		219,141,922–219,167,243	***RQCD1***
5	*+	−		98,893,720–98,901,744	***ATP5J2***
6	+	+		176,816,220–176,833,300	***DBN1***
7	*+	−	7q22.1^£^	98,874,499–98,892,932	***CPSF4***
8	+	−		218,996,724–219,022,487	***VIL1***
9	+	+	2q32.1^£^	187,916,094–188,021,266	***CALCRL***
10	+	+	2q32.2^£^	189,547,344–189,585,717	***COL3A1***
11	−	−	4p14^£^	39,874,922–39,922,676	***RHOH***
12	+	+	2q35^£^	217,245,073–217,268,517	***IGFBP5***
13	*+	−		137,648,858–137,695,415	***CDC25C***
14	*+	−	5q35.3	176,663,441–176,666,556	***PRELID1***
15	−	−	1q41^£^	212,843,155–212,904,537	***CENPF***
16	*+	−		176,761,745–176,769,183	***F12***
17	+	+		8,689,807–8,706,700	***MFAP5***
18	+	−		30,023,632–30,032,379	***GDPD3***
19	*+	−		98,994,384–99,012,013	***ZNF655***
20	*+	−	8q24.3	144,520,168–144,522,180	***C8orf51***
21	*+	−	10q21.3	69,539,196–69,641,779	***MYPN***
22	+	+	16p11.2	28,456,163–28,457,996	***NUPR1***
23	+	+		50,784,865–50,787,557	***GPR4***
24	+	+		311,432–725,606	***DIP2C***
25	*+	−	12p13.31^£^	8,646,029–8,656,706	***AICDA***
26	+	+		218,972,722–218,978,908	***CTDSP1***
27	+	−		30,102,427–30,107,898	***CORO1A***
28	+	+		38,387,813–38,445,509	***FGFR1***
29	*+	−	11q13.2^£^	67,576,902–67,645,434	***CHKA***
30	*+	−		38,007,177–38,037,040	***EIF4EBP1***
31	*+	−		780,475–786,221	*SLC25A22*
32	*+	−	10p15.3	1,075,964–1,085,061	*IDI1*
33	−	−	12p11.21	32,151,452–32,422,408	*BICD1*
34	+	+	16q24.3	88,301,042–88,314,895	*C16orf7*
35	+	+	19q13.32^£^	50,702,528–50,722,080	*VASP*
36	*−	+	20p11.2	23,007,993–23,014,977	*CD93*
37	+	−		99,402,286–99,411,623	*AZGP1*
38	*+	−	14q13.2^£^	34,291,688–34,414,604	*BAZ1A*
39	+	−		99,613,474–99,649,946	*STAG3*
40	+	−		145,624,971–145,640,620	*TONSL*
41	+	−		384,217–394,908	*PKP3*
42	+	+		29,739,288–29,766,842	*MVP*
43	+	+	8p12	37,773,932–37,820,650	*GPR124*
44	+	−		737,432–755,024	*TALDO1*
45	+	−		144,757,532–144,762,887	*PYCRL*
46	*+	−		70,385,898–70,414,285	*DDX21*
47	+	+		51,842,709–51,856,235	*DACT3*
48	+	+		29,730,910–29,734,703	*PRRT2*
49	+	+	16q22.1^£^	65,775,770–65,781,608	*EXOC3L1*
50	+	+		38256727–38263666	*AOC3*
51	*+	−		28,850,761–28,858,164	*CD19*
52	*+	−	19q13.33^£^	55,614,028–55,624,060	*SPIB*
53	+	−		50,882,558–50,887,282	*SNRPD2*
54	+	+		50,964,816–50,977,655	*DMPK*
55	+	+		51,134,631–51,168,497	*NOVA2*
56	+	+		30,488,524–30,491,229	*ZNF688*
57	+	−		145,221,948–145,224,416	*CYC1*
58	*+	−		70,650,065–70,697,321	*HKDC1*
59	*+	−		51,796,352–51,805,879	*CALM3*
60	*+	−		88,517,246–88,530,006	*TUBB3*
61	*+	−		52,033,263–52,046,043	*AP2S1*
62	+	+		51,605,929–51,608,681	*CCDC8*
63	+	−		65,790,529–65,795,428	*ELMO3*
64	*+	−		88,331,460–88,410,566	*FANCA*
65	*+	−	17q21.31^£^	38,449,840–38,530,994	*BRCA1*
66	+	−		145,707,479–145,714,008	*RECQL4*
67	*+	−		522,242–525,550	*HRAS*
68	+	+		145,619,808–145,624,735	*VPS28*
69	+	+	11p15.5	303,991–305,272	*IFITM1*
70	*+	−		99,528,340–99,537,363	*MCM7*
71	*+	−		51,969,980–51,983,653	*SLC1A5*
72	*+	−		145,714,199–145,721,365	*LRRC14*
73	+	−		551,450–554,018	*RASSF7*
74	*+	−	20p13	309,308–326,203	*TRIB3*
75	*+	−		364,124–391,187	*TBC1D20*

**In bold**, the top 30 modulators based on the highest scores on CONEXIC; Hg18: Human genome version 18 (Mar 2006 NCBI36); ^*^miRNA target prediction; positive (+) and negative (−) signs indicate the gene status with regard to the genomic gains and losses and up- or down-regulated gene expression, respectively. ^£^Regions which usually not are involved in chromosomal breakpoints.

### MicroRNA Target Prediction

Among the 75 modulators, 26 showed positive associations (genomic gain/up-regulated) and 3 showed negative associations (genomic loss/down-regulated). Inverse associations were found for 45 genes and one ORF sequence (*C8orf51*), of which 30 could be explained by miRNA regulation (Table S2). The miRNA target prediction analysis was unable to explain the inverse correlation for 15 genes.

### Modulator Characterisation

**Figure 1:**
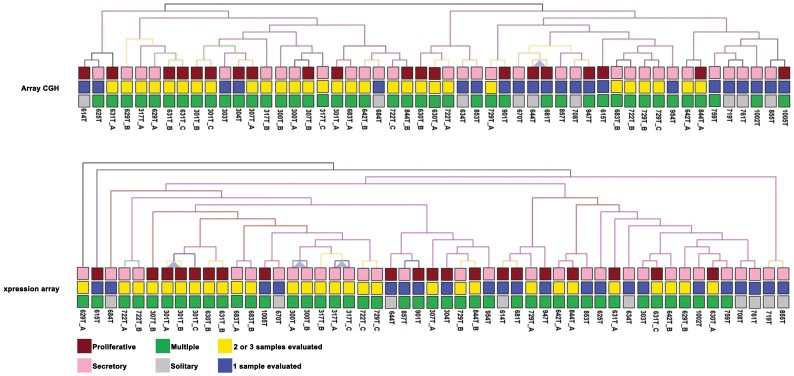
Hierarchical clustering. The patients were grouped according to the menstrual cycle phase (proliferative and secretory), number of samples evaluated and diagnosis of multiple or solitary tumours. These results show that the genomic and transcriptomic data were useful to clustering the samples regardless of the clinical features, indicating that could be markers to tumour biology (TMeV v.4.5).

Unsupervised hierarchical clustering analysis was performed to verify the association between the genomic and transcriptomic data of 75 modulators considering the number of tumours evaluated, menstrual cycle phase and diagnosis of multiple or solitary tumours. The genomic and transcriptomic profiles were not statistically associated with these clinical data (Figure 1). In addition, the distribution of the 75 modulators along the chromosomes revealed that 44 (58.66%) were mapped at regions not usually described as breakpoint targets (1p36.13, 1q41, 2q32.1, 2q32.2, 2q35, 4p14, 5q31.2, 7q22.1, 11q13.2, 12p13.31, 14q13.2, 16q22.1, 17q21.31, 19q13.32 and 19q13.33), and 23 and 5 genes (30.67% and 6.67%, respectively) mapped to telomeric or pericentromeric regions, respectively, which are frequently targets of chromosomal instability (data not shown).

### Gene Set Enrichment Analysis

Using Gene Set Enrichment Analysis (GSEA), we searched a list of up- and down-regulated genes in the ULs against 26 validated gene sets for cancer pathways from the molecular signature database (MsigDB) available at http://www.broad.mit.edu/gsea/msigdb/. The results revealed that 32 out of the 75 modulators were enriched in the 26 cancer modules (Table S3). Among these modules, *CORO1A*, *FGFR1*, *DDX21* and *DBN1* were the most frequently identified; a positive association was found for *DBN1* and *FGFR1*. The enrichment values for the 26 cancer modules were significantly associated with the 32 genes (data not shown).

### Selection of Central Modulators

The top 30 genes were selected based on the CONEXIC output ranked list of high-scoring modulators (Table 1). A positive association was found for 12 genes (*TNS1*, *HSPB7*, *DBN1*, *CALCRL*, *COL3A1*, *IGFBP5*, *MFAP5*, *NUPR1*, *GPR4*, *DIP2C*, *CTDSP1* and *FGFR1*), whereas a negative association was detected for *RHOH* and *CENPF*. The genes with a positive association were mapped to 1p36.13, 2q32.1, 2q32.2, 2q35, 5q35.3, 8p12, 10p15.3, 12p13.31, 16p11.2, and 19q13.32. The genes with a negative association were mapped to 1q41 and 4p14.

### Functional Analysis

**Figure 2:**
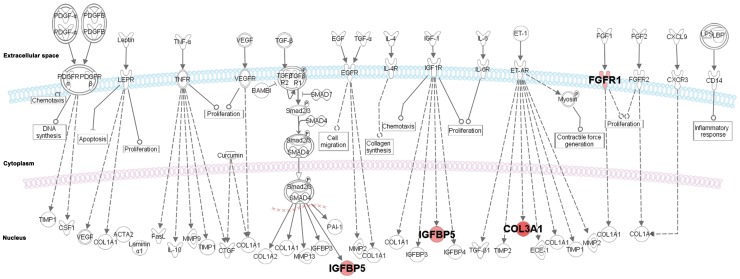
Canonical pathway from hepatic fibrosis/hepatic stellate cell activation. Cell proliferation and extracellular matrix deposition process with high similarity observed in ULs. FGFR1 and IGFBP5 were selected as potential molecular markers in ULs treatment.

The top 30 modulators were subjected to *in silico* functional analysis using Ingenuity Pathway Analysis (IPA) (Ingenuity® Systems, http://www.ingenuity.com)http://www.ingenuity.com/. We generated networks using known functions and interconnectivity of the affected genes. The modulators were present in eight gene interaction networks with scores ranging from 2 to 38 (Table S4). The analysis of the selected gene networks identified the *CALCRL, CENPF, COL3A1, FGFR, IGFBP5, GPR4, NUPR1, RHOH* and *TNS1* molecules, and the remaining pathway molecules were incorporated by IPA into the networks, as they were associated with cellular movement, development and function of the skeletal and muscular system and cell morphology (data not shown). In addition, most of the identified molecules were associated with cancer, reproductive system disease and genetic disorders (*P*<10^−4^). Cell cycle, cellular assembly and organisation and cellular growth and proliferation were the major functions associated with the dataset (*P*<10^−3^) (Table 2). Sixty-one canonical pathways were significantly associated with the modulators, although only 37 pathways were associated with the selected genes. Eight of 14 genes were associated with different canonical pathways, including the intrinsic prothrombin activation pathway (*COL3A1*), FGF signalling (*FGFR1*), ERK/MAPK signalling (*HSPB7*), VDR/RXR activation (*IGFBP5*), mTOR signalling (*RHOH*), FAK signalling (*TNS1*) and G-protein coupled receptor signalling (*CALCRL*, *GPR4*). In the canonical pathway for hepatic fibrosis/hepatic stellate cell activation, *COL3A1*, *FGFR1*, and *IGFBP5* (*P*<0.01) were involved, which indicates similarity to the biological pathways already described in ULs (Figure 2).

**Table 2:** Diseases and biological functions obtained from Ingenuity Pathway Analysis.

Diseases and Disorders	*P* value	Molecules*
Cancer	1.88^E−04^ – 4.87^E−02^	15
Reproductive System Disease	3.67^E−04^ – 4.87^E−02^	8
Genetic Disorder	6.68^E−04^ – 4.16^E−02^	9
Metabolic Disease	6.68^E−04^ – 1.53^E−02^	3
Connective Tissue Disorders	1.93^E−03^ – 3.61^E−02^	1
**Molecular and Cellular Functions**		
Cell Cycle	1.28^E−04^ – 4.72^E−02^	7
Amino Acid Metabolism	1.93^E−03^ – 1.93^E−03^	1
Cellular Assembly and Organization	1.93^E−03^ – 4.35^E−02^	7
Cellular Growth and Proliferation	1.93^E−03^ – 4.53^E−02^	10
Molecular Transport	1.93^E−03^ – 3.61^E−02^	2

* Number of molecules within each network. The molecule networks were generated from association with IPA database and our input data.

Top 30 modulators statistically associated with cancer and cell cycle, respectively.

### Interologous Interaction Database and Protein-protein Interactions

**Figure 3:**
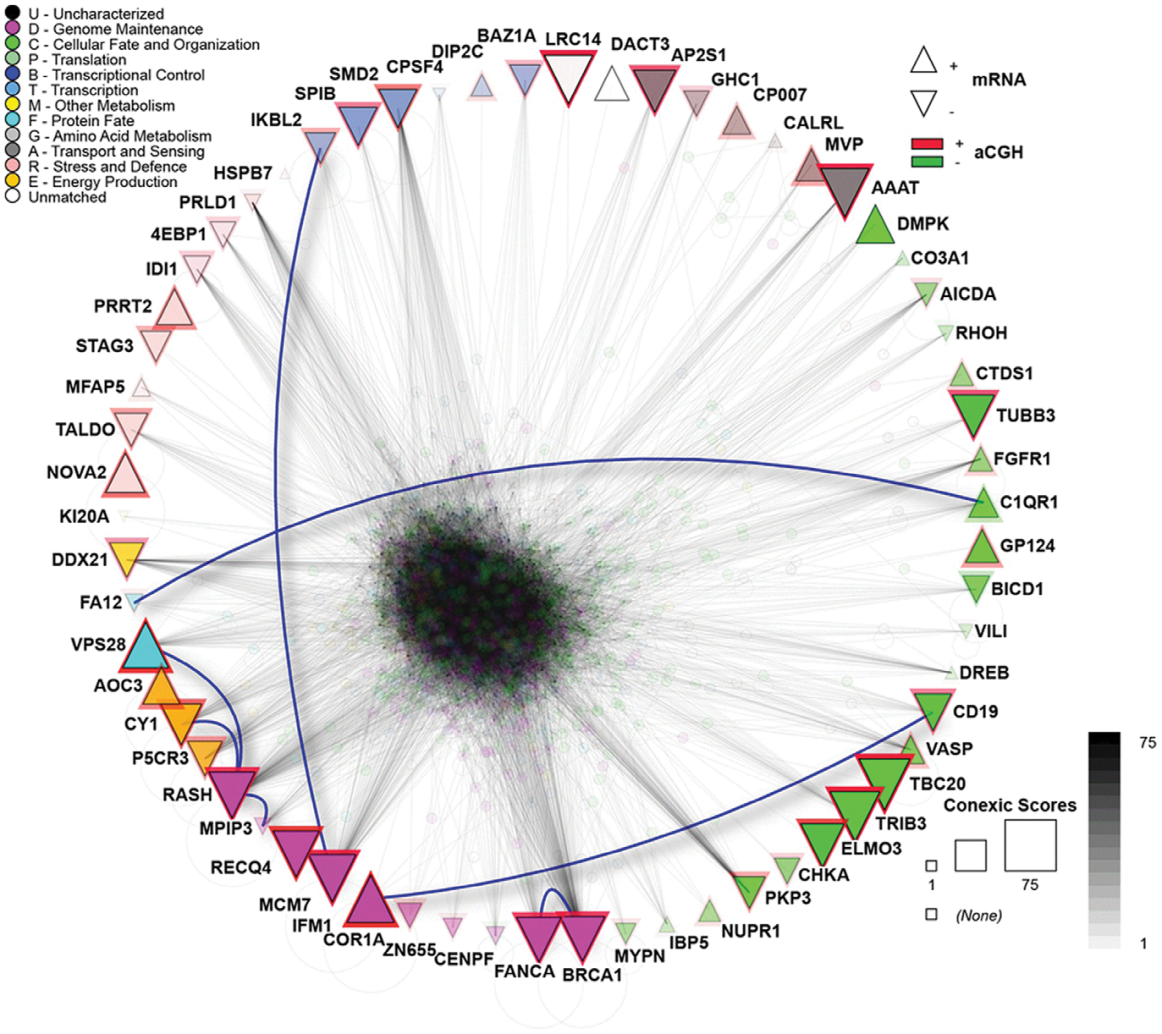
Protein-protein interaction network of 75 modulators. The 75 modulators were used to query the I2D database to obtain their interacting partners (and also interactions among the modulators). I2D v. 2.0 contained data on 62 modulators, which resulted in a large network of 1,456 proteins and 29,530 interactions. The upward triangles represent up-regulated genes, and the downward triangles represent down-regulated genes. The red and green triangle lines represent genes in amplified and deleted regions, respectively. The biological processes that the modulators are involved are represented by different colours, per the legend, and the triangle size corresponds with the score, i.e., larger triangles depict the highest scores. Direct physical interactions between modulators are represented by blue lines. The remainder of the network is partially transparent to reduce the clutter. The network visualisation and analysis was performed in NAViGaTOR 2.3.

The network generated from the 75 modulators demonstrated strong protein-protein interactions among the 62 modulators, including direct interactions between FA12 and C1QR1, IKBL2 and MCM7, COR1A and CD19, and FANCA and BRCA1 (Figure 3). Analyses were conducted using the Interologous Interaction Database (I2D) [23] to determine known and predicted interactions and using the NAViGaTOR software for network annotation, visualisation and analysis [24], as described in the methods.

### Reverse Transcription Quantitative Polymerase Chain Reaction (RT-qPCR)

**Figure 4:**
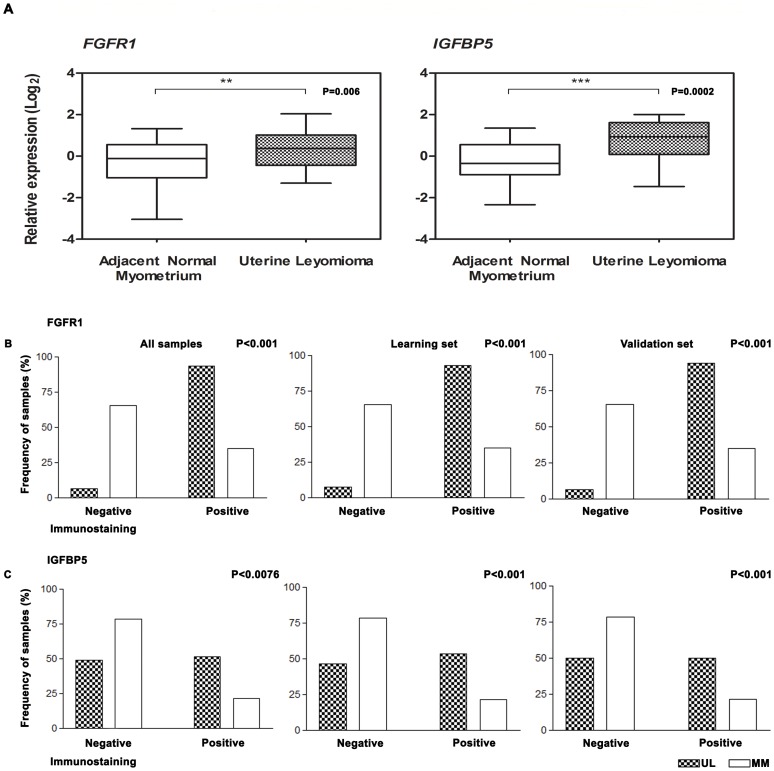
Data validation. (A) Boxplot illustrating MM (normal) and ULs (tumour) normalised to obtain relative expression values for all samples evaluated by RT-qPCR. *P* = paired *t* test significance. **P = 0.006; ***P = 0.0002; Immunostaining frequency for the (B) FGFR1 and (C) IGFBP5 proteins. The *P* values (Fisheŕs test) were obtained based on the comparison of the MM and ULs immunostaining results.

Based on previously reported findings in which *FGFR1* and *IGFBP5* stimulate cell proliferation, on their relationship as potential drug targets (Ingenuity® Systems, http://www.ingenuity.com) and on their positive association and classification among the top 30 modulators, the *FGFR1* and *IGFBP5* genes were selected for validation. Their transcripts were significant up-regulated in ULs compared with adjacent normal myometrium (MM) (*P* = 0.006 and *P* = 0.0002, respectively) (Figure 4-A). No significant association was found with respect to hormonal receptor positivity, age, skin colour, menarche age, age at first pregnancy, body index mass (BMI), menstrual cycle phase at surgery and number of tumours (data not shown).

### Immunohistochemistry (IHC)

**Figure 5:**
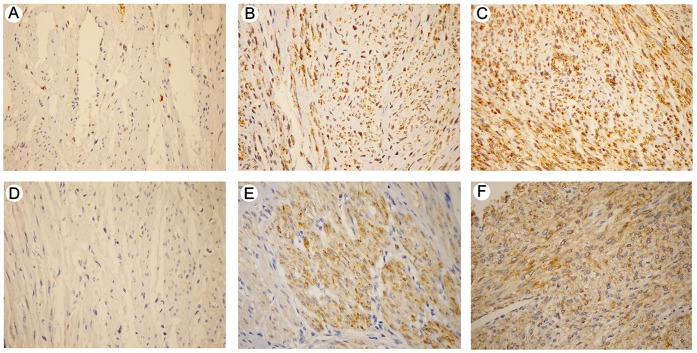
FGFR1 and IGFBP5 protein expression by immunohistochemistry in uterine leiomyomas. (A) FGFR1-adjacent normal myometrium showing FGFR1 low level expression (score 1); (B) and (C) FGFR1 cytoplasmic positive expression in uterine leiomyoma tissue (scores 2 and 3/intensity, respectively); (D) adjacent normal myometrium showing IGFBP5 negative expression (score 0); (E) and (F) IGFBP5 cytoplasmic positive expression in uterine leiomyoma tissue (scores 2 and 3/intensity, respectively) (200×).

A Spearman correlation test (Figure S1-A) revealed similar results for the different methods of analysis. The scores obtained for all ULs were plotted using the data from conventional analysis (light microscope). Positive expression was detected for the FGFR1 and IGFBP5 proteins in 93.4%, and 51.3% (All samples), of the cases, respectively, and 34.6%, and 21% (All samples), of the controls, respectively (Table S5; Figure 4-B,C, respectively). FGFR1 (Figure 5-B,C) and IGFBP5 (Figure 5-E,F) immunostaining demonstrated cytoplasmic expression. The results obtained by digital microscopy were used to perform the comparison between the immunostaining and clinical data. A significant association was found between the age at diagnosis and age at first pregnancy for the FGFR1 (*P* = 0.0211) and IGFBP5 (*P* = 0.0416), respectively (Figure S1-B), whereas increased expression of FGFR1 and IGFBP5 was more frequently identified in tumours from young patients (<40 years of age) and patients who became pregnant before 21 years of age.

## Discussion

Based on *in silico* approaches integrating genomic and transcriptomic profiles, the present study has identified candidate genes and molecular pathways associated with ULs pathogenesis, including FGFR1 and IGFBP5. These molecules have been associated with tumour proliferation and validated as therapeutic targets in breast cancer [25], [26]. However, to the best of our knowledge, this study is the first to demonstrate that *FGFR1* and *IGFBP5* amplification is associated with transcript and protein up-regulation in ULs. Thus, these molecules may be suitable candidates for therapeutic targets in ULs.

The integrative analysis using CONEXIC [19] revealed 75 modulators distributed among 25 chromosomal regions; 7 were previously described in ULs by HR-CGH or array CGH studies, including 1p36.13, 4p14, 7q22.1, 11p15.5, 14q13.2, 19q13.32 and 19q13.33 [17], [12], [27], [28], [29]. Furthermore, we also verified new genomic imbalances mapping to 1q41, 2q32.1, 2q32.2, 2q35, 5q31.2, 5q35.3, 8p12, 8q24.3, 10p15.3, 10q21.3, 11q13.2, 12p11.21, 12p13.31, 16p11.2, 16q24.3, 17q21.31, 20p11.2 and 20p13. Among the 75 modulators, only *CALCRL*, *COL3A1*, and *FGFR1* were previously described to be associated with ULs [30], [31], [32], [33]. All 75 modulators were associated with ULs pathogenesis regardless of tumour multiplicity and menstrual cycle phase, which indicates that they play central roles in the aetiology of these tumours. Recently, Hodge et al. [13] reported the transcriptional profiling of ULs according to t(12;14) and, confirmed the involvement of *HMGA2*. They also described 9 genes whose expression could distinguish the myometrial origin. These data suggest a constitutional genetic predisposition to these somatic alterations. A comparison of our 75 modulators with the top 300 genes in ULs according to t(12;14), as described by Hodge et al. [13], revealed that *DIP2C* and *PRRT2* were common in both gene lists. Based on our data, the correlation status of both genes was positive. These findings reinforce the involvement of these genes in ULs.

Of the 75 modulators, 45 demonstrated an inverse association between the array CGH and gene expression status. Similarly, in the NCI-60 cell line panel Bussey et al. [34] described negative correlation; only 42% of down-regulated genes were found in regions of DNA copy number loss. Probably, the algorithm model used by Bussey et al. [34] and by us could not be enough to reflect the CNA status/gene expression. In addition to miRNAs, other mechanisms could be involved to explain the inverse correlation found in the integrative analysis including point mutation, intra and inter-chromosomal rearrangements, expression of the second allele, epigenetic alterations, and methodologies limitations. Post-transcriptional mechanisms also have been associated with gene expression regulation, such as alternative RNA splicing, speed of transport of mRNA, and mRNA half-life. For example, RNA-binding proteins (RBPs) are able to affect every aspect in the processing of transcripts, from alternative splicing, polyadenylation, and nuclear export to cytoplasmic localization, stability, and translation [35]. Besides RBPs, miRNAs are important contributors to the post-transcriptional gene expression control. In mammals, microRNAs are predicted to control the activity of approximately 30% of all protein-coding genes, and have been shown to participate in the regulation of almost cellular process investigated [36]. In addition, the location of miRNAs at regions of genomic instability (or fragile sites) in humans has been suggested are associated with various biological phenomena, including cell growth, apoptosis, development, differentiation, and tumorigenesis [37], [38]. Gene expression regulation by miRNAs has been described in several tumours, including ULs [7], [39]. In the present study, the inverse association between the genomic/transcriptomic data could be explained by miRNA regulation for 30 of 45 genes. Moreover, as discussed above, other post-transcriptional mechanisms of gene expression regulation might be involved. Independent of the correlation status, 44 of the 75 modulators were mapped to chromosomal regions that are generally not frequent targets of breakpoints, which reinforces the relevance of these alterations in ULs development. Therefore, our data suggest that deregulated gene expression in ULs is frequently associated with genomic alterations and with the regulation of some of them by miRNA. However, further investigations using functional studies are essential to evaluate the miRNA/mRNA target regulation.

Finally, among the top 30 genes modulators that demonstrated significantly higher scores, 12 presented a positive association (*CALCRL*, *COL3A1*, *CTDSP1*, *DBN1*, *DIP2C*, *FGFR1, GPR4*, *HSPB7*, *IGFBP5*, *MFAP5*, *NUPR1*, and *TNS1*), and 2 demonstrated a negative association (*CENPF* and *RHOH*) (Table 1). The top 30 modulators were mainly associated with cancer involving the cell cycle (*P*<10^−4^) and cellular growth and proliferation (*P*<10^−3^) (Table S4).

Disruption of the cell cycle has been described in the pathogenesis of several types of tumours [40], including ULs [41]. In addition, cellular proliferation stimulated by growth factors and/or steroid hormones is one of the mechanisms associated with the increased tumour volume observed in ULs [42], [43]. The *COL3A1*, *IGFBP5* and *FGFR1* genes are directly associated with the typical features found in ULs (including fibroid formation), which strongly suggests their involvement in ULs pathogenesis. Menorrhagia is one of the most frequent symptoms associated with ULs and is characterised by excessive uterine bleeding and reduced fertility [44]. In general, prothrombin (coating factor II) activation begins with trauma to the blood vessel or exposure of the blood to collagen in a traumatised vessel wall. *COL3A1* up-regulation has been associated with increased collagen deposition in ULs [31]. Similarly, our data demonstrate a positive correlation with *COL3A1*. Previous data from our group showed that the increased expression of the *COL3A1* transcript was significant in ULs when compared with MMs (*P*<0.01) (unpublished data). The *in silico* functional analysis revealed that *COL3A1* was associated with the response to the collagenase from *Clostridium histolyticum,* which comprises a treatment approved for progressive Dupuytren contractures disease (PDCD) [45]. Because ULs and PDCD are both types of fibroproliferative disorders, *COL3A1* is thus a candidate for further studies that should evaluate its correlation with *Clostridium histolyticum* therapy and menorrhagia in UL patients.

Although the ULs present fibroid morphology, these tumours shown cell proliferation stimulated by many growth factors and steroid hormones. It has been previously described expression of fibroblast growth factor (bFGF) in the uterus, but a complete understanding of its function requires better knowledge in ULs [46]. Fibroblast growth factor receptor 1 (FGFR1), which is one of the modulators with a positive association identified in this study acts as bFGF receptor. In addition, FGFR1 transcript over-expression (*P* = 0.006) and immunoreactivity were more significantly associated with ULs when compared with MMs (*P*<0.01), regardless of the group set (Figure 4A–B). The fibroblast growth factor receptor (FGFR) family of tyrosine kinase receptors (RTKs) comprises four highly conserved members (FGFR1–4) [47]. Gain-of-function mutations and various other genetic alterations that affect the expression or activity of these FGFRs have been identified in multiple tumour types, which suggests that FGFRs are potential therapeutic targets in cancer. *FGFR1* has been described as being amplified in 10% of breast cancers [48], [49], [50]. Activated RTKs play an important role in the enhanced proliferation described in ULs. In an interesting study using proteomic approach, Yu et al. [51] reported the differential expression of RTKs in ULs in comparison with myometrial tissues. In this study, the authors described that 15 out of 17 RTKs evaluated were highly expressed in ULs, including IGF-I/IGF-IR, EGF/EGFR, FGF/FGF-R, HGF/HGF-R, and PDGF/PDGF-R gene families. In addition, *FGFR1* up-regulation was associated with menorrhagia in UL patients [32]. However, to our knowledge, *FGFR1* genomic gains have not been described in ULs. The *in silico* functional analysis showed an association between the FGFR1 molecule and pazopanib, which a tyrosine kinase inhibitor recently approved for the treatment of renal cell carcinomas (RCC) [52]. Pazopanib exerts anti-angiogenic properties via the inhibition of intracellular RTKs. The immunolocalisation of FGFR1 in ULs cells demonstrated positive expression in the cytoplasm of smooth-muscle cells [33]. Similar to our IHC results, we observed positive immunostaining in the cytoplasm of tumour cells when compared with MMs (*P*<0.001). Furthermore, the IHC results revealed that young affected patients (tumour diagnosis before 40 years of age) had an increased expression of FGFR1 in ULs (*P* = 0.0211) (Figure S1-B). These findings suggest that treatment with inhibitors of FGFR1 could be more effective in younger patients. This observation indicates that the up-regulation of *FGFR1* is important to consider for drugs that inhibit cellular growth in younger patients with ULs menorrhagia-associated.

Uterine Leiomyoma cells are also responsive to insulin-like growth factors (IGFs), which are tightly regulated by multiple factors and typically altered in cancer [53]. Insulin-like growth factor binding proteins (IGFBPs) are critical regulators of the mitogenic activity of IGFs [54]. IGFBP5 has a special nuclear transport domain, heparin-binding motif and IGF/extracellular matrix/acid-labile subunit-binding sites. Furthermore, IGFBP5 plays several functional roles in carcinogenesis and even in normal cell processes, such as cell growth, death, motility, and tissue remodelling (for review see [53]). In a non-human primate uterus, *IGFBP5* mRNA was expressed in myometrial smooth muscle cells but displayed distinctive patterns of regulation by sex steroids [55]. IGFBP5 protein expression has been reported to be a marker of poor outcome independent of the ER and PR status in patients with breast cancer [54]. According to the authors, the greatest advantage of targeting the IGF system is its crosstalk with other signalling pathways, as preclinical work has identified significant antitumor activity when IGF-1R is concordantly targeted with mTOR, ERα, the EGF receptor and HER2 in breast cancer. To the best of our knowledge, two studies reported the involvement of IGFBP5 [56], [57] in uterine leiomyomas. The aims of the Giudice et al. [56] study was to evaluate the steroid dependence of IGF, IGFBP and IGF receptor gene expression and IGFBP synthesis in ULs, using tissues from women cycling normally and that were treated with GnRHa (gonadotrophin- releasing hormone agonist). By Northern-blotting, they reported that the relative abundance of IGFBP mRNA was more significant in IGFBP4 (IGFBP4>>>IGFBP3>>IGFBP5>IGFBP2) and was not dependent on of the *in vivo* oestrogen status. Tsibris et al. [57] using gene expression arrays in ULs from 9 patients in the follicular and luteal phases of the menstrual cycle, reported 67 genes overexpressed in ULs in comparison with myometrium tissues. They described that *IGFBP5* was overexpressed in ULs (mean fold change 4.3). These findings suggest an increased activity of IGFBP5 and an oestrogen-dependent association in hormone-dependent tumours, such as ULs. Here, we have shown that the increased expression of *IGFBP5* (*P* = 0.0002) and significant cytoplasmic immunoreactivity (*P*<0.01) in the ULs when compared with MMs (Figure 4-A, C, respectively) were significantly associated with an age ≤21 y at first pregnancy (*P* = 0.0416) (Figure S1-B). These results provide additional support for the role of IGFBP5 in oestrogen-dependent tumours.

Hepatic fibrosis/hepatic stellate cell activation, which is one of the more interesting canonical pathways identified in our study, receives stimuli that could simultaneously activate *COL3A1, FGFR1*, and *IGFBP5*. Hepatic fibrosis is a chronic liver disease associated with extracellular matrix accumulation and is thus similar to the ULs pathological condition. NP603, which is an inhibitor of tyrosine kinase activity of FGFR1, inhibits the proliferation of myofibroblasts associated with liver fibrosis in rats [58]. The increased tyrosine kinase activity of FGFR1 in association with IGFBP5 in response to FGF1, IGF-1 or TGF-beta may trigger cell proliferation, whereas the increased activity of COL3A1 in response to ET-1 could lead to extracellular matrix accumulation and collagen deposition (Figure 2). Studies using *in vivo* models are crucial to assess the rate of response to drugs inhibiting that cell proliferation in ULs.

Among the identified protein-protein interactions, the most interesting finding was the interaction between the FANCA and BRCA1 proteins. Both proteins are involved in genome maintenance. The *FANCA* gene was initially associated with Fanconi anaemia, which is a recessive genetic disease, characterised by high chromosome breakage and increased sensitivity to agents that cause DNA damage and repair defects in the DNA damage [59]. Heterozygous deletions of the promoter region of this gene were associated with familial breast cancer [60]. To our knowledge, *FANCA* mutations in ULs have not been previously reported, although genetic down-regulation was identified [44], that corroborates our data. Among the patients included in our study, five presented a family history of breast cancer in first-degree relatives, but only one case presented *FANCA* and *BRCA1* alterations. Phosphorylated BRCA1 controls the downstream molecules that control the G2/M cell cycle (cdc25C, FANCA and HRAS). BRCA1 also participates in the homologous recombination process during meiosis and double-strand break repair. Therefore, the loss of activity of these molecules could result in DNA damage and contribute to the genomic instability observed in ULs.

In conclusion, the integrative analysis of the genomic and transcriptomic data provided a comprehensive and biologically meaningful insight into the tumorigenesis of ULs, thereby identifying genomic amplifications translated by the up-regulation of modulators. To our knowledge, this study is the first to use a large series of ULs evaluated by integrative genomic and transcriptomic analyses. These findings indicate that *FGFR1* and *IGFBP5* could be targets for the development of specific therapies related to cell proliferation in Uterine Leiomyomas.

## Materials and Methods

### Tissue Sample Collection

Fifty-one fresh frozen ULs and adjacent normal myometrium (MM) were collected from 34 patients who had undergone a hysterectomy procedure at the Department of Gynaecology and Obstetrics, School of Medicine, São Paulo State University, UNESP- São Paulo, Brazil between October 1995 and February 2004.

### Ethics Statement

Written informed consent from all patients was obtained during the collect period, and this series of studies was reviewed and approved by Institutional Ethics Committees of School of Medicine, São Paulo State University, UNESP - Botucatu, São Paulo, Brazil (146/2007-CEP).

### Clinical and Histopathological Parameters

Nine patients had solitary ULs, and 25 patients presented multiple tumours. In 13 patients, one UL was evaluated, and in 7 and 5 patients, 2 and 3 ULs were investigated, respectively. All women were premenopausal with regular menstrual cycles and had not received exogenous hormones or hormone suppression therapy for at least three months before the surgery. At surgery, 13 patients were in the proliferative menstrual cycle phase, and 21 were in the secretory period. The medical records were examined in 2012 to retrieve the clinical and pathological data. The ages of the patients ranged from 35 to 51 years, with a mean age of 45 years. All tumours were histopathologically diagnosed as typical ULs.

### Genomic and Transcriptomic Data

#### Array comparative genomic hybridisation and data analysis

Genomic DNA from 51 ULs was isolated by the standard procedure using sodium dodecyl sulphate/proteinase K digestion followed by phenol-chloroform extraction and ethanol precipitation. The samples were treated with 20 µg/mL RNaseA (Sigma-Aldrich, St. Louis, MO, USA). High-quality genomic DNA (500 ng) from the cases and a reference (male commercial genomic DNA) (Promega, Madison, WI, USA) were hybridizised on Agilent Human 4×44K CGH Microarrays (Agilent Technologies, Santa Clara, CA, USA) according to the manufacturer’s instructions. After scanning the slides (Agilent scanner at a 5-µm resolution), the array data were extracted using the default CGH settings of the Agilent Feature Extraction Software (version 10.1.1.1) (Agilent Technologies). The CNAs analyses were performed using a segmented genomic dataset (DNAcopy, http://www.bioconductor.org/packages/2.3/bioc/html/DNAcopy.html) and the identification of significant targets in cancer (JISTIC) algorithm [61]. JISTIC uses a smoothed log ratio to compute a statistical G-score, which represents the aberration intensity of each probe. The obtained G-score is compared with that expected by chance using a permutation test, and the significant results are obtained using a *q*-value under a threshold (0.25) corrected by the False Discovery Rate (FDR) [62]. The JISTIC results were displayed using the Integrative Genomic Viewer (IGV) [63].

#### Gene expression microarrays and data analysis

Fifty-one samples evaluated by array-CGH were also investigated by gene expression microarrays. Total RNA was extracted from frozen tissues (ULs and normal myometrium) using the RNeasy Mini Kit (QIAGEN, Hilden, Germany) according to the manufacturer's instructions.

The microarray experiments were performed using Two-Color Human GE 4×44K Microarrays (Agilent Technologies). Isolated RNA (500–1000 ng) was converted to cDNA with reverse transcriptase and an oligo_(dT)_ primer bearing a T7 promoter, followed by *in vitro* transcription with T7 RNA polymerase to create amplified antisense RNA. The microarray image analysis was performed using the Agilent Feature Extraction Software (version 10.1.1.1) (Agilent Technologies). The statistical analysis was performed using background-corrected mean signal intensities from each dye channel. The microarray data were normalised by intensity-dependent global normalisation (LOWESS) using the Agilent Feature Extraction Software (v.10.1.1.1). Both the genomic and transcriptomic data discussed in this publication have been deposited in NCBI's Gene Expression Omnibus and are accessible through GEO Series accession number GSE43027 (http://www.ncbi.nlm.nih.gov/geo/query/acc.cgi?acc=GSE43027) and GSE42939 (http://www.ncbi.nlm.nih.gov/geo/query/acc.cgi?acc=GSE42939).

#### Significant genes based on expression analysis

The raw data were normalised by median-centring the genes for each array and then log_2_ transformed. Additionally, a filter was applied to remove probes with low reproducibility. The SAM method was applied to identify differential probes [64]. FDR <0.05 was used to determine the significance threshold for genes and limit the likelihood of type I errors [65], [66]. To select significant genes, we used threshold values of log_2_ratio ≥1.0 and ≤ –1.0 fold change to classify the genes as up- or down-regulated, respectively. Hierarchical clustering analysis (HCL) was performed using the Complete Linkage method with Pearson correlation (TMeV v.4.5).

### Integrative Analysis

The algorithm CONEXIC was used to assess the association between DNA copy number alterations and changes in the transcript abundance of genes within defined regions [19], [67]. The algorithm CONEXIC is inspired by Module Networks [66], but has been augmented by a number modification that makes it suitable for identifying drivers. This approach is based on a score-guided search to identify the combination of modulators that best explains the behaviour of a gene expression module across tumour samples and searches for those with the highest score within the amplified or deleted region. The resulting output is a ranked list of high-scoring modulators that both correlate with differences in gene expression modules across samples and are located in amplified or deleted regions in a significant number of these samples. The fact that the modulators are amplified or deleted indicates that they are likely to control the expression of the genes in the corresponding modules. Because the modulators are altered in a significant number of tumours, it is reasonable to assume that the modulator provides an advantage to the tumour.

### MicroRNA Target Prediction

Samples where the association between gene copy number and transcript level (genomic gain/down-expression or genomic loss/over-expression) failed (indicating inverse association), the miRNA regulation might be one potential reason. The TargetScan (http://www.targetscan.org.br), PicTar (http://pictar.mdc-berlin.de/cgi-bin/PicTar_vertebrate.cgi) and mirDIP (http://ophid.utoronto.ca/mirDIP) *in silico* tools were used to predict the miRNA targets.

### Gene Set Enrichment Analysis

GSEA was applied to determine whether a differentially expressed gene shows significant over-representation when compared with specific functional pathways [67], [68]. We only considered validated pathways obtained from MSigDB (http://www.broadinstitute.org/gsea/msigdb/index.jsp; MSigDB c2 GO category) and those derived from cancer studies obtained from PubMed.

### *In silico* Functional Analysis

Ingenuity Pathway Analysis (IPA v8.0, Ingenuity® Systems, Redwood City, CA, USA; http://www.ingenuity.com) was used to identify the canonical pathways and biological interaction networks of genes obtained from the integrative analysis. Fischer’s exact test was applied to identify significant functions, networks and pathways represented within the respective gene sets. This program displayed a score – log(*P*-value) that represented the probability of finding genes in networks and pathways relative to other molecules and assembled the molecules into specific network/pathways based on random chance.

### Interologous Interaction Database and Protein-protein Interactions

Additional *in silico* analysis was performed using known and predicted physical protein-protein interactions from I2D version 2.0 (http://ophid.utoronto.ca/i2d) [23]. Visualisation and analysis of the resulting network were conducted in NAViGaTOR version 2.3 (http://ophid.utoronto.ca/navigator) [24]. Briefly, to generate a list of the target proteins, we extracted all of the interacting partners from I2D and mapped the interactions among them. The resulting network was annotated, visualised and analysed in NAViGaTOR. The final figure was exported in the SVG format and finalised in Adobe Illustrator with legends.

## Data Validation

### Reverse Transcription Quantitative Polymerase Chain Reaction

Complementary DNA (cDNA) synthesis was performed as previously described [69]. Fifty-one UL samples were evaluated by array CGH and gene expression microarray simultaneously, and an additional 26 samples (n = 77) matched with the adjacent normal myometrium were investigated by RT-qPCR. The primer sets for the validation (*FGFR1* and *IGFBP5)* and reference (*ACTB*, *GAPDH*, *GUSB*, *HPRT* and *RPLP0)* genes were designed using Primer-Blast online software (http://www.ncbi.nlm.nih.gov/tools/primer-blast/). The primers were designed to flank the same region detected by the gene expression microarray (Table S6). The PCR amplifications were performed by robotic pipetting using QIAgility (QIAGEN, Courtaboeuf, France) in a total volume of 12.5 µL that contained the Power SYBR Green PCR Master Mix (Applied Biosystems; Foster City, CA, USA), 20 ng of cDNA and each primer at a concentration of 200 nM. All samples were analysed in duplicate and submitted to the following cycle conditions: initial incubation at 95°C for 10 min, followed by 40 cycles at 95°C for 15s, and 60°C for 1 min, followed by a dissociation curve in a 7500 Real time PCR System (Applied Biosystems). The quantitative data were analysed using Sequence Detection System software v1.0 (Applied Biosystems).

### Tissue Microarray (TMA) Construction

Core biopsies were taken using a Tissue Microarrayer (Beecher Instruments®, Silver Springs, USA). Tissue cores (1.0 mm) from each specimen were punched and arrayed in duplicate on a recipient paraffin block with 0.2 mm of spacing. Adhesives were coated for subsequent UV cross-linkage (Instrumedics Inc®, Hackensack, NJ). Slides were dipped in a layer of paraffin to prevent oxidation, and kept in a −20°C freezer. Paraffin-embedded ULs were cut (3 µm) and mounted on silane-coated glass slides for hematoxylin-eosin staining and immunohistochemistry reaction.

### Immunohistochemistry

In total, 104 ULs and 32 MMs from 81 patients were arranged on a tissue microarray TMA; among them, 51 were also investigated using CGH arrays and gene expression microarrays. The sections, mounted on glass slides and dried for 30 min at 37°C, were deparaffinised in xylene and rehydrated in a series of graded alcohols. Endogenous peroxidase activity was blocked by incubating the sections in a methanol bath containing 3% hydrogen peroxide for 20 min, followed by washing in distilled water. All sections were initially submitted to heat-induced epitope retrieval using a citrate buffer (pH 9.0). The FGFR1 (GeneTex, San Antonio, TX, USA; clone polyclonal; dilution 1∶400) and IGFBP5 (Santa Cruz, Santa Cruz, CA, USA; clone C18; dilution 1∶100) antibodies were incubated for 30 minutes at room temperature. After the primary antibody washed with phosphate-buffered saline (PBS), the slides were incubated with the Ventana detection system (Ventana, Tucson, AZ, USA) for 30 min. A ready-to-use DAB (3,3′-diaminobenzidine) solution was applied for 5 minutes to each section and removed by rinsing with distilled water. The slides were counterstained with haematoxylin, dehydrated in ethanol, cleared in xylene and mounted using Entelan (Merck, Darmstadt, Germany). All reactions were processed in a Benchmark autostainer (Ventana, Tucson, AZ, USA). The immunostaining was scored as negative (score 0) or positive (1, 2 and 3) by two independent investigators (M.A.C.D and R.M.R.) according to its intensity using light and digital microscopes (ScanScope, Aperio, Vista, CA, USA).

## Statistical Analysis

ANOVA or *t*-test analysis was applied to compare the transcript levels and clinic-pathological characteristics. The correlation analyses for the gene expression were performed using a Spearman’s rank test. The samples evaluated by IHC were grouped in a *learning set* (samples used for genomic and transcriptomic experiments, n = 51) and a *validation set* (additional samples, n = 28). The Spearman correlation was applied for two IHC methods of analysis: conventional and automated. Fisher’s test (comparison between ULs and MMs), *t*-test or ANOVA (comparison between the IHC scores and clinical data) was applied in the comparisons between the variables. The mean transcript quantification and protein score were considered for multiple tumours. A 5% significance level was used for all tests. The statistical analyses were conducted using GraphPad Prism v5.0 (GraphPad Software Inc., La Jolla, CA, USA) and SPSS v17.0 (SPSS; Chicago, IL, USA) for Windows.

## Supporting Information

Figure S1 Immunohistochemistry analysis. (A) Spearman correlation between two techniques for capturing immunostaining images using a light and a digital microscope. (B) FGFR1 and IGFBP5 increased expression statistically associated with age (ANOVA) and age at first pregnancy (*t* test) among young ULs patients, respectively.
(TIF)

Table S1 Recurrent copy number alterations identified by JISTIC among 51 Uterine Leiomyomas samples.
(DOC)

Table S2 miRNA target prediction analysis from genes identified on integrative analysis that showed an inverse association between genomic and transcriptomic data.
(DOC)

Table S3 Genes identified on cancer module genes obtained from Gene Set Enrichment Analysis – GSEA.
(DOC)

Table S4 Ingenuity Pathways Analysis (IPA) networks from top 30 modulators.
(DOC)

Table S5 Immunohistochemistry analysis for FGFR1 and IGFBP5 proteins in Uterine Leiomyomas and adjacent normal myometrium samples.
(DOC)

Table S6 Primer sequences and properties for all transcripts evaluated.
(DOC)
