# Supplementary figures and images for: An Integrative Genomic and Transcriptomic Analysis Reveals Potential Targets Associated with Cell Proliferation in Uterine Leiomyomas
Source: PLoS One. 2013 Mar 4;8(3):e57901. doi: 10.1371/journal.pone.0057901 (PMC3587425; doi:10.1371/journal.pone.0057901)

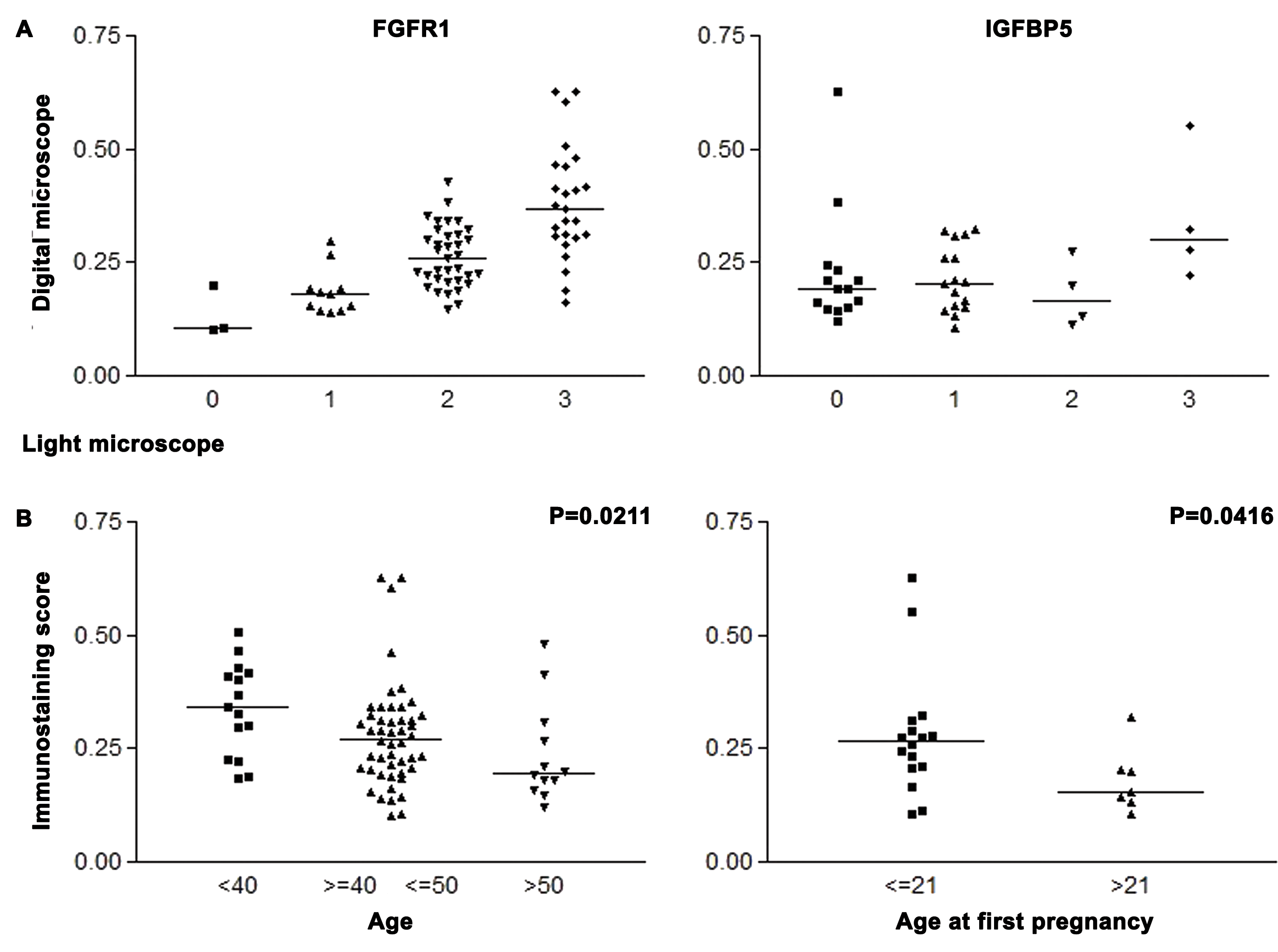

Supplement: Figure S1 — Immunohistochemistry analysis. (A) Spearman correlation between two techniques for capturing immunostaining images using a light and a digital microscope. (B) FGFR1 and IGFBP5 increased expression statistically associated with age (ANOVA) and age at first pregnancy (t test) among young ULs patients, respectively. (TIF) [file pone.0057901.s001.tif]
