# Supplementary material for: An Integrative Genomic and Transcriptomic Analysis Reveals Potential Targets Associated with Cell Proliferation in Uterine Leiomyomas
Source: PLoS One. 2013 Mar 4;8(3):e57901. doi: 10.1371/journal.pone.0057901 (PMC3587425; doi:10.1371/journal.pone.0057901)
Supplement: Table S1 — Recurrent copy number alterations identified by JISTIC among 51 Uterine Leiomyomas samples. (DOC) [file pone.0057901.s002.doc]

**Table S1.** Recurrent copy number alterations identified by JISTIC among 51 Uterine Leiomyomas samples.

| **Chromosome** | **Position** | **Size (bp)** | **Event** |
| --- | --- | --- | --- |
| 1p36.13 | 16210136-16404475 | 194339 | Gain |
| 1q31.3 | 193404142-194937836 | 1533694 | Loss |
| 1q41 | 212893807-214486070 | 1592263 | Loss |
| 2q32.1-q32.2 | 187336585-189552794 | 2216209 | Gain |
| 2q35 | 217253192-219351693 | 2098501 | Gain |
| 4p14 | 39121392-40671431 | 1550039 | Loss |
| 4q13.1 | 63649592-66171072 | 2521480 | Loss |
| 4q28.3 | 132475881-135650424 | 3174543 | Loss |
| 5p13.3 | 32031565-32459137 | 427572 | Gain |
| 5q31.1-q31.2 | 134274907-135585827 | 1310920 | Loss |
| 5q31.2 | 137509066-137894706 | 385640 | Gain |
| 5q35.3 | 176628647-176902434 | 273787 | Gain |
| 7q22.1 | 98882914-102068500 | 3185586 | Gain |
| 7q31.33 | 124268700-124352498 | 83798 | Loss |
| 7q36.1 | 150157293-150323899 | 166606 | Gain |
| 8p12-p11.23 | 37606006-38685823 | 1079817 | Gain |
| 8q22.3-q23.1 | 105119923-108955123 | 3835200 | Gain |
| 8q24.3 | 143934407-144604977 | 670570 | Gain |
| 8q24.3 | 144723137-146027963 | 1304826 | Gain |
| 9p13.3 | 33871385-34349039 | 477654 | Gain |
| 9p13.3 | 34380035-34602962 | 222927 | Gain |
| 9p12-p11.2 | 42014069-42702421 | 688352 | Gain |
| 9q34.3 | 137569888-138038551 | 468663 | Loss |
| 9q34.3 | 138467512-138845556 | 378044 | Gain |
| 10p15.3 | 0-1167989 | 1167989 | Gain |
| 10q21.3 | 69249439-70694950 | 1445511 | Gain |
| 11p15.5 | 209012-838551 | 629539 | Gain |
| 11q13.1-q13.2 | 66956644-67906757 | 950113 | Gain |
| 11q14.1 | 82287494-82844323 | 556829 | Gain |
| 12p13.31 | 7620638-8938808 | 1318170 | Gain |
| 12p11.21 | 31233552-32196110 | 962558 | Loss |
| 12q24.11 | 109668284-109732691 | 64407 | Gain |
| 13q31.1 | 79882431-84123782 | 4241351 | Loss |
| 14q13.2 | 34245057-34584738 | 339681 | Gain |
| 14q32.33 | 104210820-104323543 | 112723 | Gain |
| 16p13.3 | 154832-649034 | 494202 | Gain |
| 16p13.12-p13.11 | 14674167-15423013 | 748846 | Loss |
| 16p11.2 | 28184420-30915100 | 2730680 | Gain |
| 16p11.2-p11.1 | 34059589-34739434 | 679845 | Loss |
| 16q22.1 | 65732841-65799873 | 67032 | Gain |
| 16q22.1 | 65977069-66551504 | 574435 | Gain |
| 16q23.1 | 74665577-75131336 | 465759 | Loss |
| 16q24.3 | 88147752-88526617 | 378865 | Gain |
| 17p13.1-p12 | 10029176-12411350 | 2382174 | Gain |
| 17q21.2 | 36917868-36931811 | 13943 | Gain |
| 17q21.31 | 38117300-39051741 | 934441 | Gain |
| 17q25.3 | 76857866-77619424 | 761558 | Gain |
| 18q22.1-q22.2 | 61548752-64944845 | 3396093 | Loss |
| 19p13.3 | 1173033-1426319 | 253286 | Gain |
| 19p13.2 | 11723538-12106305 | 382767 | Loss |
| 19q13.32 | 50340704-52381739 | 2041035 | Gain |
| 19q13.32-q13.33 | 53504306-54293549 | 789243 | Gain |
| 19q13.33 | 54838449-55064096 | 225647 | Gain |
| 19q13.33 | 55617858-55704434 | 86576 | Gain |
| 20p13 | 0-512804 | 512804 | Gain |
| 20p11.21 | 22971225-23206367 | 235142 | Loss |
| 20q12 | 37240929-38699013 | 1458084 | Loss |
| 21p11.1 | 10013263-10117957 | 104694 | Loss |
| 21p11.1 | 10013263-10117957 | 104694 | Gain |
| 22q13.33 | 49041686-49401541 | 359855 | Gain |

bp=base pairs.
