# Supplementary material for: An Integrative Genomic and Transcriptomic Analysis Reveals Potential Targets Associated with Cell Proliferation in Uterine Leiomyomas
Source: PLoS One. 2013 Mar 4;8(3):e57901. doi: 10.1371/journal.pone.0057901 (PMC3587425; doi:10.1371/journal.pone.0057901)
Supplement: Table S2 — miRNA target prediction analysis from genes identified on integrative analysis that showed an inverse association between genomic and transcriptomic data. (DOC) [file pone.0057901.s003.doc]

**Table S2.** miRNA target prediction analysis from genes identified on integrative analysis that showed an inverse association between genomic and transcriptomic data.

| **Gene**  **symbol** | **TargetScan** | **PicTar** |
| --- | --- | --- |
| *AICDA* | *hsa-miR-155* | *hsa-miR-155* |
| *AP2S1* | *-* | *hsa-miR-34b, hsa-miR-34c, hsa-miR-34a* |
| *ATP5J2* | *-* | *-* |
| *AZGP1* | *-* | *-* |
| *BAZ1A* | *hsa-miR-137* | *-* |
| *BRCA1* | *hsa-miR-218* | *hsa-miR-197, hsa-miR-143, hsa-miR-205, hsa-miR-132, hsa-miR-370, hsa-miR-30a-3p, hsa-miR-140, hsa-miR-185, hsa-miR-154* |
| *C8orf51* | ND | ND |
| *CALM3* | *hsa-miR-22* | *hsa-miR-122a, hsa-miR-22, hsa-miR-27b, hsa-miR-27a, hsa-miR-196b, hsa-miR-196a, hsa-miR-29c, hsa-miR-29b, hsa-miR-29a, hsa-miR-320,* |
| *CD19* | *-* | *-* |
| *CDC25C* | *hsa-miR-767-3p* | *-* |
| *CD93* | *hsa-miR-216a* | *-* |
| *CHKA* | *hsa-miR-30e, hsa-miR-30a, hsa-miR-30d, hsa-miR-30b, hsa-miR-30c* | *-* |
| *CORO1A* | *-* | *-* |
| *CPSF4* | *hsa-miR-214* | *hsa-miR-214, hsa-miR-23a, hsa-miR-23b, hsa-let-7c, hsa-let-7g, hsa-let-7b, hsa-let-7f, hsa-let-7i, hsa-let-7a, hsa-miR-98, hsa-let-7e, hsa-let-7d* |
| *CYC1* | *-* | *-* |
| *DDX21* | *hsa-miR-607* | *-* |
| *EIF4EBP1* | *hsa-miR-125b, hsa-miR-125a-5p* | *hsa-miR-125b, hsa-miR-125a* |
| *ELMO3* | *-* | *-* |
| *F12* | *hsa-miR-330-3p* | *-* |
| *FANCA* | *-* | *hsa-miR-26a, hsa-miR-26b* |
| *GDPD3* | *-* | *-* |
| *HKDC1* | *hsa-miR-876-5p, hsa-miR-1243* | *-* |
| *HRAS* | *hsa-miR-892a* | *-* |
| *IDI1* | *hsa-miR-570* | *-* |
| *KIF20A* | *hsa-miR-153* | *hsa-miR-369, hsa-miR-374* |
| *LRRC14* | *-* | *hsa-miR-370, hsa-miR-28, hsa-miR-122a* |
| *MCM7* | *hsa-miR-519b-3p, hsa-miR-519a, hsa-miR-519c-3p, hsa-miR-548p* | *-* |
| *MYPN* | *hsa-miR-214* | *-* |
| *NFKBIL2* | *-* | *-* |
| *PKP3* | *-* | *-* |
| *PRELID1* | *hsa-miR-22* | *-* |
| *PYCRL* | *-* | *-* |
| *RASSF7* | *-* | *-* |
| *RECQL4* | *-* | *-* |
| *RQCD1* | ND | ND |
| *SLC1A5* | *hsa-miR-125a-5p, hsa-miR-125b* | *hsa-miR-122a, hsa-miR-137* |
| *SLC25A22* | *hsa-miR-613, hsa-miR-1, hsa-miR-206* | *hsa-miR-296, hsa-miR-1, hsa-miR-206, hsa-miR-337* |
| *SNRPD2* | *-* | *-* |
| *SPIB* | *hsa-miR-1299, hsa-miR-520a-5p, hsa-miR-525-5p* | *hsa-miR-146, hsa-miR-218, hsa-miR-328* |
| *STAG3* | *-* | *-* |
| *TALDO1* | *-* | *-* |
| *TBC1D20* | *hsa-miR-150* | *-* |
| *TRIB3* | *hsa-miR-24* | *-* |
| *TUBB3* | *hsa-miR-429, hsa-miR-200c, hsa-miR-200b* | *-* |
| *VIL1* | *-* | *-* |
| *ZNF655* | *hsa-miR-181c, hsa-miR-181a, hsa-miR-181b, hsa-miR-181d* | *-* |

*TargetScan* (<http://www.targetscan.org/>); *PicTar* (http://pictar.mdc-berlin.de/cgi-bin/PicTar_vertebrate.cgi); ND=genes not identified on databases; (-) no miRNA prediction.
