# Supplementary material for: An Integrative Genomic and Transcriptomic Analysis Reveals Potential Targets Associated with Cell Proliferation in Uterine Leiomyomas
Source: PLoS One. 2013 Mar 4;8(3):e57901. doi: 10.1371/journal.pone.0057901 (PMC3587425; doi:10.1371/journal.pone.0057901)
Supplement: Table S3 — Genes identified on cancer module genes obtained from Gene Set Enrichment Analysis – GSEA. (DOC) [file pone.0057901.s004.doc]

**Table S3.** Genes identified on cancer module genes obtained from Gene Set Enrichment Analysis – GSEA.

| **Gene Set Name** | **Genes in Gene Set (K)** | **Genes in Overlap (k)** | **k/K** | ***P* value** |
| --- | --- | --- | --- | --- |
| module_53 module_3 | 42 | *CENPF, CORO1A, AOC3* | 0.0714 | <10^-3^ |
| module_8 | 406 | *CORO1A, MCM7, SLC1A5, DDX21, NUPR1, DBN1, EIF4EBP1, VIL1, CHKA* | 0.0222 | <10^-3^ |
| module_509 | 14 | *AP2S1, IDI1* | 0.1429 | <10^-3^ |
| module_315 | 15 | *CENPF, TUBB3* | 0.1333 | <10^-3^ |
| module_5 | 423 | *CORO1A, AOC3, DDX21, NUPR1, FGFR1, CALCRL, COL3A1, MVP* | 0.0189 | <10^-2^ |
| module_292 | 133 | *IFITM1, SPIB, CD19, STAG3* | 0.0301 | <10^-2^ |
| module_3 | 375 | *MCM7, SLC1A5, DDX21, DBN1, EIF4EBP1, FGFR1, CALCRL* | 0.0187 | <10^-2^ |
| module_118 | 395 | *CENPF, CORO1A, DDX21, DBN1, EIF4EBP1, FGFR1, RECQL4* | 0.0177 | <10^-2^ |
| module_86 | 43 | *HRAS, RHOH* | 0.0465 | <10^-2^ |
| module_137 | 531 | *CORO1A, NUPR1, DBN1, EIF4EBP1, FGFR1, HRAS, NOVA2, BICD1* | 0.0151 | <10^-2^ |
| module_12 | 351 | *DDX21, NUPR1, DBN1, FGFR1, COL3A1, DIP2C* | 0.0171 | <10^-2^ |
| module_9 | 114 | *EIF4EBP1, VIL1, HRAS* | 0.0263 | <10^-2^ |
| module_430 | 52 | *CORO1A, FANCA* | 0.0385 | <10^-2^ |
| module_1 | 361 | *AOC3, DDX21, NUPR1, DBN1, FGFR1, COL3A1* | 0.0166 | <10^-2^ |
| module_105 | 194 | *CHKA, IDI1, FGFR1, COL3A1* | 0.0206 | <10^-2^ |
| module_57 | 54 | *MCM7, CDC25C* | 0.0370 | <10^-2^ |
| module_345 | 122 | *IFITM1, SPIB, CD19* | 0.0246 | <10^-2^ |
| module_254 | 58 | *NUPR1, IFITM1* | 0.0345 | <10^-2^ |
| module_337 | 60 | *CENPF, DDX21* | 0.0333 | <10^-2^ |
| module_53 | 391 | *CENPF, CORO1A, DDX21, DBN1, EIF4EBP1, RECQL4* | 0.0153 | <10^-2^ |
| module_129 | 217 | *DBN1, FGFR1, HRAS, NOVA2* | 0.0184 | <10^-2^ |
| module_119 | 138 | *SPIB, CD19, STAG3* | 0.0217 | <10^-2^ |
| module_122 | 138 | *AOC3, COL3A1, MFAP5* | 0.0217 | <10^-2^ |
| module_308 | 67 | *CORO1A, CDC25C* | 0.0299 | <10^-2^ |
| module_438 | 67 | *CENPF, CORO1A* | 0.0299 | <10^-2^ |
| module_32 | 232 | *SNRPD2, EIF4EBP1, TUBB3, RHOH* | 0.0172 | <10^-1^ |

GSEA - <http://www.broadinstitute.org/gsea/index.jsp>. Computational cancer modules (C4.CM) of MSigDB. K=overlapping modulators associated with modules; k=differentially expressed genes associated with modulators; k/K=ratio between genes on modules.
