# Supplementary material for: An Integrative Genomic and Transcriptomic Analysis Reveals Potential Targets Associated with Cell Proliferation in Uterine Leiomyomas
Source: PLoS One. 2013 Mar 4;8(3):e57901. doi: 10.1371/journal.pone.0057901 (PMC3587425; doi:10.1371/journal.pone.0057901)
Supplement: Table S4 — Ingenuity Pathways Analysis (IPA) networks from top 30 modulators. (DOC) [file pone.0057901.s005.doc]

**Table S4**. Ingenuity Pathways Analysis (IPA) networks from top 30 modulators.

| **Network** | **Molecules in Network** | **Score** | **Focus Molecules** | **Top Functions** |
| --- | --- | --- | --- | --- |
| 1 | **↓AICDA**, Akt, **↑CALCRL**, CDC14A, **↓CDC25C**, **↓CENPF**, **↓CHKA**, **↑COL3A1**, **↓CORO1A**, EDNRA, **↓EIF4EBP1**, ERK1/2, **↓F12**, F Actin, **↑FGFR1**, **↑GPR4**, GRB7, Histone h3, Igf, **↑IGFBP5**, LOX, MSK1/2, NFkB (complex), **↑NUPR1**, PI3K (complex), PLK3, PNO1, PPM1B, PTGER3, RAMP3, **↓RHOH**, SMOC2, **↑TNS1**, Vegf, **↓VIL1** | 38 | 16 | Cellular Movement, Skeletal and Muscular System Development and Function, Cell Morphology |
|  |  |  |  |  |
| 2 | Actin, ACTR3, ATP5A1, ATP5F1, **↓ATP5J2**, C12orf11, CDC14A, **↑DBN1**, EIF4G1, FBP1, FLNA, HNF4A, HNRNPH1, **↑HSPB7**, **↓KIF20A**, KPNB1, MAP2K6, MAP3K3, MAPK9, MYB, PNO1, PPP1CA, PRPF4, RIPK3, RPLP1, **↓RQCD1**, RUVBL2, SPHK1, SUPT5H, TCF12, TRAF6, TRIM37, WNK1, YWHAB, **↓ZNF655** | 12 | 6 | Developmental Disorder, Neurological Disease, DNA Replication, Recombination, and Repair |
|  |  |  |  |  |
| 3 | **↑DIP2C**, PCBD1 | 3 | 1 | Genetic Disorder, Metabolic Disease, Organ Development |
|  |  |  |  |  |
| 4 | **↓PRELID1**, STAT6 | 3 | 1 | Cell-mediated Immune Response, Cellular Development, Cellular Function and Maintenance |
|  |  |  |  |  |
| 5 | Cpsf, **↓CPSF4**, FIP1L1, MARK3 | 2 | 1 | RNA Post-Transcriptional Modification, Cancer, Infection Mechanism |
|  |  |  |  |  |
| 6 | DLL1, JAG1, JAG2, **↑MFAP5**, NOTCH1 | 2 | 1 | Nervous System Development and Function, Tissue Morphology, Gene Expression |
|  |  |  |  |  |
| 7 | CDCA3, **↑CTDSP1**, mir-124, POLR2A, SMAD1, SNAI1 | 2 | 1 | Embryonic Development, Tissue Development, Cellular Growth and Proliferation |
|  |  |  |  |  |
| 8 | ACTN2, ACTN3, ANKRD1, ANKRD23, **↓MYPN**, NEB, NEBL, PPP1CA, TTN | 2 | 1 | Cell Morphology, Cellular Assembly and Organization, Cellular Function and Maintenance |

**In bold**, molecules on focus from dataset; arrows above indicate genes with positive association; arrows below indicate genes with negative association.
