# Supplementary material for: An Integrative Genomic and Transcriptomic Analysis Reveals Potential Targets Associated with Cell Proliferation in Uterine Leiomyomas
Source: PLoS One. 2013 Mar 4;8(3):e57901. doi: 10.1371/journal.pone.0057901 (PMC3587425; doi:10.1371/journal.pone.0057901)
Supplement: Table S5 — Immunohistochemistry analysis for FGFR1 and IGFBP5 proteins in Uterine Leiomyomas and adjacent normal myometrium samples. (DOC) [file pone.0057901.s006.doc]

**Table S5**. Immunohistochemistry analysis for FGFR1 and IGFBP5 proteins in Uterine Leiomyomas and adjacent normal myometrium samples.

| **Data set** | **Protein** | **Samples** | **Frequency of immunostaining**  **N (%)** | | | |
| --- | --- | --- | --- | --- | --- | --- |
|  |  |  | **0** | **1** | **2** | **3** |
| **All cases** | **FGFR1** | UL | 5 (6.58) | 11 (14.47) | 35 (46.05) | 25 (32.89) |
|  |  | MM | 17 (65.38) | 7 (26.92) | 1 (3.85) | 1 (3.85) |
|  | **IGFBP5** | UL | 39 (48.75) | 22 (27.50) | 8 (10) | 11 (13.75) |
|  |  | MM | 22 (78.57) | 1 (3.57) | 3 (10.71) | 2 (7.14) |
| **Learning set** | **FGFR1** | UL | 2 (7.14) | 2 (7.14) | 14 (50) | 10 (35.71) |
|  |  | MM | 17 (65.38) | 7 (26.92) | 1 (3.85) | 1 (3.85) |
|  | **IGFBP5** | UL | 13 (46.43) | 9 (32.14) | 3 (10.71) | 3 (10.71) |
|  |  | MM | 22 (79.57) | 1 (3.57) | 3 (10.71) | 2 (7.14) |
| **Validation set** | **FGFR1** | UL | 3 (6.25) | 9 (18.75) | 21 (43.75) | 15 (31.25) |
|  |  | MM | 17 (65.38) | 7 (26.92) | 1 (3.85) | 1 (3.85) |
|  | **IGFBP5** | UL | 26 (50) | 13 (25) | 5 (9.62) | 8 (15.38) |
|  |  | MM | 22 (79.57) | 1 (3.57) | 3 (10.71) | 2 (7.14) |

UL= Uterine Leiomyomas; MM = Adjacent Normal Myometrium.
