# Supplementary material for: An Integrative Genomic and Transcriptomic Analysis Reveals Potential Targets Associated with Cell Proliferation in Uterine Leiomyomas
Source: PLoS One. 2013 Mar 4;8(3):e57901. doi: 10.1371/journal.pone.0057901 (PMC3587425; doi:10.1371/journal.pone.0057901)
Supplement: Table S6 — Primer sequences and properties for all transcripts evaluated. (DOC) [file pone.0057901.s007.doc]

**Table S6.** Primer sequences and properties for all transcripts evaluated.

| **Gene symbol** | **Accession number** | **Primer sequences** | **Amplicon length** |
| --- | --- | --- | --- |
| *ACTB** | NM_001101.3 | F - 5'TTCCTGGGCATGGAGTC3' | 84bp |
|  |  | R - 5'CAGGTCTTTGCGGATGTC3' |  |
| *GAPDH** | NM_002046 | F - 5'TGCACCACCAACTGCTTAG3' | 176bp |
|  |  | R - 5'GATGCAGGGATGATGTTC3' |  |
| *GUSB** | NM_000181.3 | F - 5'GAAAATACGTGGTTGGAGAGCTCATT3' | 101bp |
|  |  | R - 5'CCGAGTGAAGATCCCCTTTTTA3' |  |
| *HPRT1** | NM_000194.2 | F - 5'TCATTATGCTGAGGATTTGGAAAG3' | 101bp |
|  |  | R - 5'GGCCTCCCATCTCCTTCATC3' |  |
| *RPLP0** | NM_001002.3 | F - 5'GGAGACGCATTACACCTTC3' | 139bp |
|  |  | R - 5'CTTCACCTTAGCTGGGG3' |  |
| *COL3A1* | NM_000090.3 | F -5'TGGTCCTCCTGGTAGTAATGGTA3' | 158bp |
|  |  | R - 5'ATCCCTTCTCTCCTGGTTGGC3' |  |
| *FGFR1* | NM_023110.2 | F - 5'ACCGTGTGACCAAAGTGGCTGT3' | 155bp |
|  |  | R - 5'CAAGGGACCATCCTGCGTGC3' |  |
| *IGFBP5* | NM_000599.3 | F - 5'gcagtgcaaaccttcccgt3' | 81bp |
|  |  | R - 5'tccatgcctggcagcttc3' |  |

F = forward primer; R = reverse primer; bp = base pairs; * Reference gene.
